# Differential Transcriptomic Profile of *Piscirickettsia salmonis* LF-89 and EM-90 During an *In Vivo* Spatial Separation Co-Culture in Atlantic Salmon

**DOI:** 10.3390/microorganisms12122480

**Published:** 2024-12-02

**Authors:** Gabriela Carril, Hanne C. Winther-Larsen, Marie Løvoll, Henning Sørum

**Affiliations:** 1 Department of Paraclinical Sciences, Faculty of Veterinary Medicine, Norwegian University of Life Sciences, 1433 Ås, Norway; 2 Section for Pharmacology and Pharmaceutical Biosciences, Department of Pharmacy, University of Oslo, 0316 Oslo, Norway; h.c.winther-larsen@farmasi.uio.no; 3 VESO Aqualab, 7810 Namsos, Norway; marie.lovoll@veso.no

**Keywords:** *Salmo salar*, SRS, piscirickettsiosis, intracellular pathogen, bacterial co-culture, RNA-seq

## Abstract

Salmonid rickettsial septicemia (SRS) is a critical sanitary problem in the Chilean aquaculture industry since it induces the highest mortality rate in salmonids among all infectious diseases. *Piscirickettsia salmonis*, a facultative intracellular bacterium, is the biological agent of SRS. In Chile, two genogroups of *P. salmonis*, designated as LF-89 and EM-90, have been identified. Previous studies suggested that their cohabitation triggers the expression of virulence effectors, which may be related to a higher pathogenicity in salmonids during co-infection with both *P. salmonis* genogroups. Therefore, we aimed to evaluate if the physical contact between two isolates from LF-89 and EM-90 is necessary to activate this effect. Through a spatially separated *in vivo* co-culture inside Atlantic salmon (*Salmo salar*) post smolts and RNA-seq analysis, we compared the differentially expressed genes (DEGs) with previous results from an *in vivo* mixed co-culture. The results showed that although the LF-89-like isolate and the EM-90-like isolate had a similar DEG profile under both co-culture conditions, important virulence factors observed during the mixed co-cultures (i.e., flagellar-related genes, CydD, and NCS2) were absent in the spatially separated co-cultures. Hence, the synergistic effect linked to increased pathogenicity to the host may be driven by the physical co-localization and contact between the *P. salmonis* LF-89-like and EM-90-like isolates.

## 1. Introduction

*Piscirickettsia salmonis* is a Gram-negative facultative intracellular bacterium and a member of the Gamma-proteobacteria class that causes salmonid rickettsia septicemia (SRS) in salmonids such as Atlantic salmon (*Salmo salar*), Coho salmon (*Oncorhynchus kisutch*) and rainbow trout (*Oncorhynchus mykiss*) [1]. SRS is the most severe bacterial disease in Chilean salmon farming, inducing high fish mortality [2] and important economic losses for aquaculture [3]. Although there are commercial vaccines available against *P. salmonis* [4], they have low efficacy (considering industry expectations) and must be complemented with antibiotic treatments to control SRS [5,6]. Thus, a deeper biological knowledge of *P. salmonis* and its host–pathogen interaction is essential to improve SRS management.

Following comprehensive studies based on genetic variability [7,8,9,10], phenotypic differences [11,12], and pathologic outcomes [13,14], two *P. salmonis* genogroups, defined as LF-89 and EM-90, have been described in Chile. In addition, a separate third genogroup (NC) has been found in *P. salmonis* isolates from Norway and Canada [15,16,17], where SRS has a minimal impact on fish morbidity and mortality.

In Chile, it has been demonstrated that SRS can be caused by co-infection with *P. salmonis* LF-89 and EM-90 in farmed Atlantic salmon, where both genogroups have been detected at the farm, fish, and tissue levels [18]. Moreover, in a previous study, we demonstrated that the cohabitation of *P. salmonis* LF-89 and EM-90 genogroups can modulate their virulence under *in vivo* co-culture conditions [19], which can be related to an increased pathogenicity to the host.

The reported effects of bacterial co-infections on fish include altered pathogen prevalence and population dynamics and increased pathogen virulence [20]. This highlights the importance of studying the interaction between *P. salmonis* LF-89 and EM-90 in salmonids. Co-infections can contribute to higher fish mortality and to the development and persistence of diseases [21], as recently described during an *in vivo* challenge with *P. salmonis* genogroups in Atlantic salmon post-smolts [22].

Therefore, the aim of this study was to evaluate whether the synergistic effect related to the expression of virulence factors linked to a higher pathogenicity to the host was driven by the physical co-localization of both *P. salmonis* genogroups. To evaluate this, we followed a similar approach to that employed by Carril et al. [19], with an *in vivo* co-culture in Atlantic salmon, but using a spatial separation co-culture system [23]. This method involved the *in vivo* growth of two isolates of *P. salmonis* LF-89 and EM-90 independently contained within dialysis tubes placed inside the host body [24] to prevent direct physical contact between them, but allowing an indirect exchange of metabolites and secreted molecules for communication.

## 2. Materials and Methods

**Figure 1:**
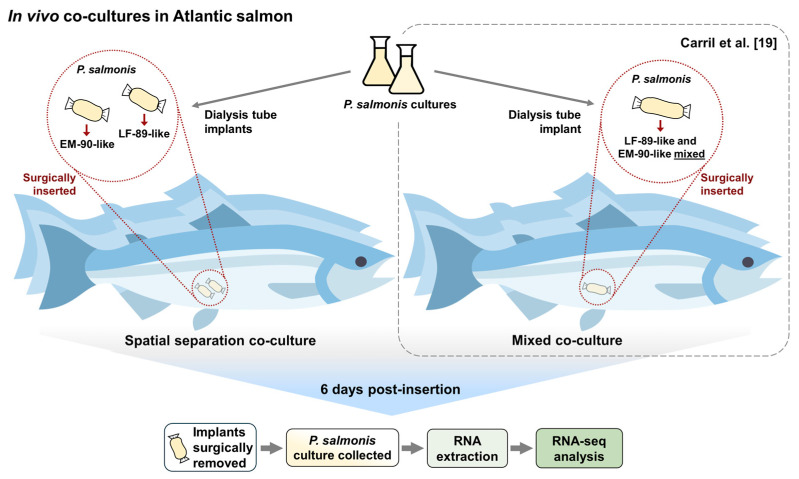
Methodology workflow of the experimental design (Carril et al. [19]).

Atlantic salmon post smolts (~1 kg), maintained in seawater facilities at the Norwegian Institute for Water Research (Solbergstrand, Norway), were used in this study after the approval of Mattilsynet (FOTS ID 26316) following the laws and regulations for experiments in the EU (Directive 2010/637EU) and Norway (FOR-2015-06-18-761). Specifically, fish were kept at 12.5 °C (in triplicate), and two implants containing individual isolates from *P. salmonis* genogroups (LF-89 or EM-90) were inserted into each abdominal cavity (Figure 1). *P. salmonis* isolates (Psal-013: LF-89-like, and Psal-182: EM-90-like) were obtained from the head kidney and liver of Atlantic salmon in Chile during 2012 and 2018, respectively.

For bacterial cultures, *P. salmonis* isolates were grown in FN2 broth (incubated at 18 °C with shaking) until the logarithmic growth phase started (OD600nm of 0.2). Then, the implants were prepared using 20 cm long and 25 mm diameter dialysis tubes (12–14 kDa molecular weight cut-off, Spectrum Laboratories Inc., Rancho Dominguez, CA, USA) knotted at one end and autoclaved in PBS 1X. For the implant’s inoculation, three sterile dialysis tubes were filled with 2.5 mL of Psal-013 bacterial culture, and three with 2.5 mL of Psal-182 culture and knotted again for closing. Thereafter, following Carril et al. [19], fish were anesthetized in a water bath of 0.005% benzocaine (Benzoak VET, Euro-Pharma, Leknes, Norway), and through a 4 cm incision, two dialysis tubes (one of each *P. salmonis* culture) were put inside each fish for the spatially separated *in vivo* co-cultures. The incisions were sewn along with three to four sutures through all layers of the tissue [24] and the fish were returned to the tank for recovery. After 6 days (before any disease symptoms could be detected), fish were euthanized with a benzocaine overdose (Benzoak VET, 200 mg mL^−1^). The implants were removed and washed with PBS 1X. Finally, the bacterial culture from each dialysis tube was collected and stored on ice until further use

Total RNA was extracted aseptically from each bacterial culture using Qiagen RNeasy Mini Kit (Qiagen, Venlo, The Netherlands), followed by rRNA depletion using QIAseq FastSelect 5S/16S/23S (Qiagen). Library preparation and RNA sequencing were performed at the Norwegian Sequencing Center (UiO, Oslo, Norway) using TruSeq Stranded mRNA library prep and Illumina NovaSeq SP System (150 bp paired-end RNA sequencing). Raw RNA-seq files were processed using Orion High-Performance Computing Cluster at the Norwegian University of Life Sciences [25]. Here, raw reads were trimmed using BBDuk (v34.56), followed by alignment with the *P. salmonis* genome Psal-013 (ASM970875v1—RefSeq GCF_009708755.1) or Psal-182 genome (ASM970941v1—RefSeq GCF_009709415.1) using HISAT (v2.1.0). The fragments mapped were counted with featureCounts (v1.4.6-1) and differentially expressed genes (DEGs) between groups were estimated using SARTools (v1.7.3) package in Rstudio (v4.2.0). Significant DEGs were determined when the adjusted *p*-value (padj) was <0.05. Data were visualized using GraphPad Prism (v8.0.1).

## 3. Results

Data from RNA-seq analyses from each pairwise comparison are shown in Table 1. In addition, DEGs distribution with full metadata (normalized gene expression, description, and statistical analysis) is listed in Table S1.

**Figure 2:**
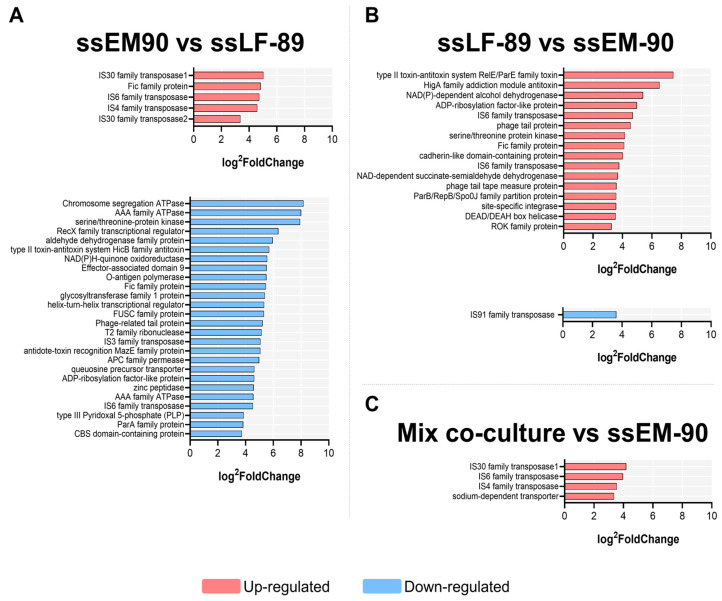
Differential transcriptomic profile of the *P. salmonis* LF-89-like (Psal-013) and EM-90-like (Psal-182) isolates during the spatial separation (ss) *in vivo* co-culture in Atlantic salmon. (**A**) ssEM-90 vs. ssLF-89. (**B**) ssLF-89 vs. ssEM-90. (**C**) Mixed co-culture vs. ssEM-90. Hypothetical proteins (HP) are not shown (details in Table S1). Data are sorted by log^2^FoldChange. Up-regulated genes in red, Down-regulated genes in blue.

**Figure 3:**
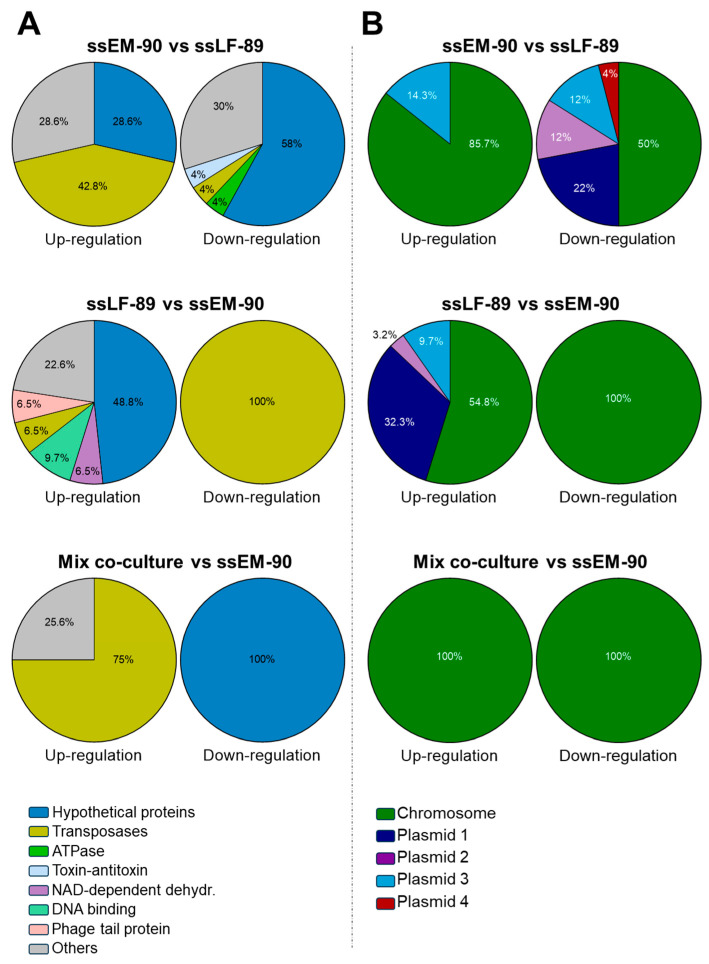
Classification of DEGs found during spatial separation co-culture in Atlantic salmon (in percentage of total). (**A**) Main gene descriptions. (**B**) DEG origin on the chromosome or plasmids.

The EM-90-like spatially separated culture (ssEM-90), compared to the LF-89-like spatially separated culture (ssLF-89), showed seven up-regulated genes (Table 1), of which four were related to a transposase family (Figure 2A). Specifically, two were related to IS30 (5.03-fold and 3.38-fold), one to IS4 (4.59-fold), and one to IS6 (4.73-fold). In addition, the origin of six of these DEGs was the chromosome and one was a plasmid (Figure 2B). Also, ss-EM-90 showed fifty down-regulated genes, where twenty-four were hypothetical proteins (with no known functions), while the rest were related to the regulation of cell proliferation (Figure 3A). The top five of these down-regulated genes were two AAA ATPases (8.18-fold and 8.01-fold), serine/threonine-protein kinase (7.94-fold), RecX family transcriptional regulator (6.36-fold), aldehyde dehydrogenase family protein (5.96-fold), and the type II toxin-antitoxin system HicB family antitoxin (5.70-fold). In this case, 50% of DEGs corresponded to the chromosome and the other 50% to plasmids (Figure 3B).

Regarding ssLF-89 compared to ssEM-90 (Figure 2B), thirty-one up-regulated genes were found. Overall, 54.6% of these belonged to the chromosome of LF-89-like and 45.5% to plasmids (Figure 3B). Fifteen DEGs were hypothetical proteins (Figure 3A), and from the rest, the first five most expressed genes were the type II toxin-antitoxin system RelE/ParE family toxin (7.45-fold), HigA family addiction module antitoxin (6.52-fold), NAD(P)-dependent alcohol dehydrogenase (5.40-fold) (all three from plasmids), ADP-ribosylation factor-like protein (4.98-fold), and the IS6 family transposase (4.70-fold). There was only one down-regulated gene (IS91 family transposase, 3.60-fold).

The pairwise comparison of the mixed co-culture to the ssEM-90 culture (Figure 2C) showed four up-regulated DEGs, and two down-regulated DEGs. From the up-regulated genes, the three first correspond respectively to the genes of IS30 family transposase (4.188-fold), IS6 family transposase (3.965-fold), and IS4 family transposase (3.538-fold), located in the chromosome. In contrast, the two down-regulated genes were identified as hypothetical proteins. Lastly, the corresponding analysis of the mixed co-culture with the ssLF-89 culture, showed no DEGs in the pairwise comparison. 

## 4. Discussion

The use of *in vivo* cultivation in which semi-permeable tubes containing cultures of *P. salmonis* LF-89-like or EM-90-like were surgically placed in the abdominal cavity of Atlantic salmon allowed us to evaluate how the pathogens respond to the host environment, since the bacterial cultures were communicating with the host through pores big enough to allow molecular trafficking, while avoiding active infection of the eukaryotic cells. Also, to evaluate the requirement of direct physical contact between *P. salmonis* LF-89-like and EM-90-like isolates to induce a differential virulence factor expression, our current work discusses the data from the spatially separated co-culture system compared to a previous study with mixed *in vivo* co-cultures [19].

Interestingly, the ssEM-90-like co-culture showed a similar DEG profile to what was found in the EM-90-like isolate during the mixed co-culture and monoculture [19], but with a smaller number of up-regulated transposases. Specifically, four transposase genes were found to be significantly up-regulated in both conditions (IS3, IS4, IS6, and IS30 family transposase). This suggests that contact with the LF-89-like isolate is unnecessary to trigger this expression in the EM-90-like isolate. However, the contact between *P. salmonis* genogroups could increase the gene expression modulation in the bacteria. Due to the crucial role of transposases in driving evolutionary processes [26], this finding supports the idea of the ongoing speciation of the EM-90 genogroup, and the unprecedentedly high number of transposases found in *P. salmonis* genomes [17]. In addition, these mobile elements produce DNA rearrangements, which can regulate transcriptional processes [27,28], helping bacteria to avoid host defenses by altering their surface antigens [29]. In the *P. salmonis* EM-90-like isolate, this adaptation could allow the bacteria to avoid detection by the host immune system, increasing its capacity to survive during the early infection process. Nevertheless, further research must be conducted to evaluate this hypothesis.

Moreover, our results showed that the plasmids found in *P. salmonis* are a relevant source of virulence factors and transposases, since they were differentially expressed in response to co-culture. According to Olasz et al. [30], the transposition of IS30 in plasmids and chromosomes induces a complex transposition network within the bacterial population, maintained through a cycle of rearrangements. This network leads to variability in subsequent bacterial generations, suggesting the importance of plasmids for *P. salmonis*’s development of virulence, which has been described previously by Saavedra et al. [31].

In the ssLF-89-like co-culture four up-regulated virulence factors were found to be shared by previous analysis on the mixed *in vivo* co-culture [19]. Specifically, the type II toxin-antitoxin system RelE/ParE, HigA module antitoxin, NAD(P)-dependent alcohol dehydrogenase, and cadherin-like domain-containing protein. This could be a specific response to the cohabitation with the *P. salmonis* EM-90-like isolate, and likely communication through the dialysis pores in the case of the spatial separated co-culture, due to their absence in the LF-89-like *in vivo* monoculture [19]. 

The expression of the genes encoding the flagellar hook-associated protein flgK, the thiol reductant ABC exporter subunit CydD, and the NCS2 family permease (all up-regulated by LF-89-like and EM-90-like isolates during mixed co-cultures [19]) was absent during spatial separation co-cultures. Flgk is related to cell adhesion/invasion [32], while cydD has a key role in maintaining redox homeostasis and the disulfide bond folding of periplasmic and secreted proteins, which is critical for bacterial virulence [33]. NCS2 has nucleobase transmembrane transporter activity, which has been reported to contribute to virulence in *Francisella tularensis* attenuated mutants [34]. Thus, the physical co-localization and contact between both *P. salmonis* LF-89-like and EM-90-like isolates could have a synergistic effect by enhancing the up-regulation of virulence factors in both genogroups. This can boost *P. salmonis* colonization and pathogenicity in Atlantic salmon during co-infection.

## Figures and Tables

**Table 1:** Differentially expressed genes (DEGs) in *P. salmonis* during spatial separation co-cultures in Atlantic salmon (vs: versus). Up: Up-regulated DEGs. Down: Down-regulated DEGs.

Pairwise Comparison	Up	Down
ssEM-90 vs. ssLF-89 ^a^	7	50
ssLF-89 vs. ssEM-90 ^b^	31	1
Mixed co-culture vs. ssEM-90 ^c^	4	2
Mixed co-culture vs. ssLF-89 ^d^	0	0

^a^ DEGs found in ssEM-90 relative to ssLF-89, by alignment with the *P. salmonis* Psal-182 genome (ASM970941v1—RefSeq GCF_009709415.1). ^b^ DEGs found in ssLF-89 relative to ssEM-90, by alignment with the *P. salmonis* Psal-013 genome (ASM970875v1—RefSeq GCF_009708755.1). ^c^ DEGs found in Mixed co-culture relative to ssEM-90, by alignment with the *P. salmonis* Psal-182 genome (ASM970941v1—RefSeq GCF_009709415.1). ^d^ DEGs found in Mixed co-culture relative to ssLF-89, by alignment with the *P. salmonis* Psal-013 genome (ASM970875v1—RefSeq GCF_009708755.1).

## Data Availability

The data supporting this study can be found in Gene Expression Omnibus-NCBI (GSE266847).

## Supplementary Materials

The following supporting information can be downloaded at: https://www.mdpi.com/article/10.3390/microorganisms12122480/s1, Supplemental Table S1. DEGs list results with metadata from RNA-seq analysis.

## References

[1] Fryer J.L., Hedrick R.P. (2003). *Piscirickettsia salmonis*: A Gram-negative intracellular bacterial pathogen of fish. J. Fish Dis..

[2] Sernapesca (2013). Informe con Antecedentes Sanitarios de Agua Dulce y Mar.

[3] Maisey K., Montero R., Christodoulides M. (2017). Vaccines for piscirickettsiosis (salmonid rickettsial septicaemia, SRS): The Chile perspective. Expert Rev. Vaccines.

[4] SAG Productos Biológicos Inmunológicos con Registro Provisional uso en Salmónidos 2024. https://www.sag.gob.cl/sites/default/files/lista_salmonidos_registro_provisional_02072024.pdf.

[5] Figueroa C., Torrealba D., Morales-Lange B., Mercado L., Dixon B., Conejeros P., Silva G., Soto C., Gallardo J.A. (2022). Commercial Vaccines Do Not Confer Protection Against Two Genogroups of *Piscirickettsia salmonis*, LF-89 and EM-90, in Atlantic Salmon. Biology.

[6] Valenzuela-Aviles P., Torrealba D., Figueroa C., Mercado L., Dixon B., Conejeros P., Gallardo-Matus J. (2022). Why vaccines fail against Piscirickettsiosis in farmed salmon and trout and how to avoid it: A review. Front. Immunol..

[7] Bohle H., Henriquez P., Grothusen H., Navas E., Sandoval A., Bustamante F., Bustos P., Mancilla M. (2014). Comparative Genome Analysis of Two Isolates of the Fish Pathogen *Piscirickettsia salmonis* from Different Hosts Reveals Major Differences in Virulence-Associated Secretion Systems. Genome Announc..

[8] Bravo C., Martinez V. (2016). Whole-genome comparative analysis of the pathogen *Piscirickettsia salmonis*. Vet. Microbiol..

[9] Nourdin-Galindo G., Sanchez P., Molina C.F., Espinoza-Rojas D.A., Oliver C., Ruiz P., Vargas-Chacoff L., Carcamo J.G., Figueroa J.E., Mancilla M. (2017). Comparative Pan-Genome Analysis of *Piscirickettsia salmonis* Reveals Genomic Divergences within Genogroups. Front. Cell. Infect. Microbiol..

[10] Isla A., Saldarriaga-Cordoba M., Fuentes D.E., Albornoz R., Haussmann D., Mancilla-Schulz J., Martinez A., Figueroa J., Avendano-Herrera R., Yanez A. (2019). Multilocus sequence typing detects new *Piscirickettsia salmonis* hybrid genogroup in Chilean fish farms: Evidence for genetic diversity and population structure. J. Fish Dis..

[11] Otterlei A., Brevik O.J., Jensen D., Duesund H., Sommerset I., Frost P., Mendoza J., McKenzie P., Nylund A., Apablaza P. (2016). Phenotypic and genetic characterization of *Piscirickettsia salmonis* from Chilean and Canadian salmonids. BMC Vet. Res..

[12] Saavedra J., Hernandez N., Osses A., Castillo A., Cancino A., Grothusen H., Navas E., Henriquez P., Bohle H., Bustamante F. (2017). Prevalence, geographic distribution and phenotypic differences of *Piscirickettsia salmonis* EM-90-like isolates. J. Fish Dis..

[13] Rozas-Serri M., Ildefonso R., Pena A., Enriquez R., Barrientos S., Maldonado L. (2017). Comparative pathogenesis of piscirickettsiosis in Atlantic salmon (*Salmo salar* L.) post-smolt experimentally challenged with LF-89-like and EM-90-like *Piscirickettsia salmonis* isolates. J. Fish Dis..

[14] Rozas-Serri M., Pena A., Arriagada G., Enriquez R., Maldonado L. (2018). Comparison of gene expression in post-smolt Atlantic salmon challenged by LF-89-like and EM-90-like *Piscirickettsia salmonis* isolates reveals differences in the immune response associated with pathogenicity. J. Fish Dis..

[15] Reid H.I., Griffen A.A., Birkbeck T.H. (2004). Isolates of *Piscirickettsia salmonis* from Scotland and Ireland show evidence of clonal diversity. Appl. Environ. Microbiol..

[16] Tandberg J.I., Lagos L.X., Langlete P., Berger E., Rishovd A.L., Roos N., Varkey D., Paulsen I.T., Winther-Larsen H.C. (2016). Comparative Analysis of Membrane Vesicles from Three *Piscirickettsia salmonis* Isolates Reveals Differences in Vesicle Characteristics. PLoS ONE.

[17] Schober I., Bunk B., Carril G., Freese H.M., Ojeda N., Riedel T., Meier-Kolthoff J.P., Goker M., Sproer C., Flores-Herrera P.A. (2023). Ongoing diversification of the global fish pathogen *Piscirickettsia salmonis* through genetic isolation and transposition bursts. ISME J..

[18] Rozas-Serri M., Pena A., Gardner I., Penaloza E., Maldonado L., Munoz A., Mardones F.O., Rodriguez C., Ildefonso R., Senn C. (2023). Co-Infection by LF-89-Like and EM-90-Like Genogroups of *Piscirickettsia salmonis* in Farmed Atlantic Salmon in Chile: Implications for Surveillance and Control of Piscirickettsiosis. Pathogens.

[19] Carril G., Winther-Larsen H.C., Løvoll M., Sørum H. (2023). Cohabitation of *Piscirickettsia salmonis* genogroups (LF-89 and EM-90): Synergistic effect on growth dynamics. Front. Cell. Infect. Microbiol..

[20] Kinnula H., Mappes J., Sundberg L.R. (2017). Coinfection outcome in an opportunistic pathogen depends on the inter-strain interactions. BMC Evol. Biol..

[21] Sundberg L.R., Ketola T., Laanto E., Kinnula H., Bamford J.K., Penttinen R., Mappes J. (2016). Intensive aquaculture selects for increased virulence and interference competition in bacteria. Proc. Biol. Sci..

[22] Carril G., Morales-Lange B., Løvoll M., Inami M., Winther-Larsen H.C., Øverland M., Sørum H. (2024). Salmonid Rickettsial Septicemia (SRS) disease dynamics and Atlantic salmon immune response to *Piscirickettsia salmonis* LF-89 and EM-90 co-infection. Vet. Res..

[23] Kapoore R.V., Padmaperuma G., Maneein S., Vaidyanathan S. (2022). Co-culturing microbial consortia: Approaches for applications in biomanufacturing and bioprocessing. Crit. Rev. Biotechnol..

[24] Colquhoun D.J., Sørum H.N. (1998). Outer membrane protein expression during *in vivo* cultivation of Vibrio salmonicida. Fish Shellfish Immun..

[25] Morales-Lange B., Agboola J.O., Hansen J.O., Lagos L., Oyas O., Mercado L., Mydland L.T., Øverland M. (2021). The Spleen as a Target to Characterize Immunomodulatory Effects of Down-Stream Processed *Cyberlindnera jadinii* Yeasts in Atlantic Salmon Exposed to a Dietary Soybean Meal Challenge. Front. Immunol..

[26] Gebrie A. (2023). Transposable elements as essential elements in the control of gene expression. Mobile DNA.

[27] De Palmenaer D., Siguier P., Mahillon J. (2008). IS4 family goes genomic. BMC Evol. Biol..

[28] Lysnyansky I., Calcutt M.J., Ben-Barak I., Ron Y., Levisohn S., Methe B.A., Yogev D. (2009). Molecular characterization of newly identified IS3, IS4 and IS30 insertion sequence-like elements in *Mycoplasma bovis* and their possible roles in genome plasticity. FEMS Microbiol. Lett..

[29] Siguier P., Gourbeyre E., Chandler M. (2014). Bacterial insertion sequences: Their genomic impact and diversity. FEMS Microbiol. Rev..

[30] Olasz F., Szabó M., Veress A., Bibó M., Kiss J. (2022). The dynamic network of IS30 transposition pathways. PLoS ONE.

[31] Saavedra J., Grandon M., Villalobos-Gonzalez J., Bohle H., Bustos P., Mancilla M. (2018). Isolation, Functional Characterization and Transmissibility of p3PS10, a Multidrug Resistance Plasmid of the Fish Pathogen *Piscirickettsia salmonis*. Front. Microbiol..

[32] Li P., Zong W., Zhang Z., Lv W., Ji X., Zhu D., Du X., Wang S. (2023). Effects and molecular mechanism of flagellar gene flgK on the motility, adhesion/invasion, and desiccation resistance of *Cronobacter sakazakii*. Food Res. Int..

[33] Holyoake L.V., Poole R.K., Shepherd M. (2015). The CydDC Family of Transporters and Their Roles in Oxidase Assembly and Homeostasis. Adv. Microb. Physiol..

[34] Meibom K.L., Charbit A. (2010). *Francisella tularensis* metabolism and its relation to virulence. Front. Microbiol.

